# Fault-Tolerant Control of Magnetically-Levitated Rotor with Redundant Structures Based on Improved Generalized Linearized EMFs Model

**DOI:** 10.3390/s21165404

**Published:** 2021-08-10

**Authors:** Baixin Cheng, Xin Cheng, Shao Song, Shuai Deng, Rougang Zhou, Yefa Hu, Huachun Wu

**Affiliations:** 1 School of Mechanical & Electronic Engineering, Wuhan University of Technology, Wuhan 430070, China; chengbaixin@126.com (B.C.); chengx@whut.edu.cn (X.C.); dengshuai2014@163.com (S.D.); huyefa@whut.edu.cn (Y.H.); whc@whut.edu.cn (H.W.); 2 Hubei Maglev Engineering Technology Research Center, Wuhan University of Technology, Wuhan 430070, China; 3 China Ship Development and Design Center, Wuhan 430070, China; songshao1986@126.com; 4 School of Mechanical Engineering, Hangzhou Dianzi University, Hangzhou 310002, China

**Keywords:** magnetic bearings, redundant structures, fault-tolerant control, electromagnetic force

## Abstract

Fault tolerance is one of the effective methods to improve the reliability of magnetic bearings, and the redundant magnetic bearing provides a feasible measure for fault-tolerant control. The linearization and accuracy of the electromagnetic force (EMF) from the redundant structures is crucial for designing fault-tolerant controllers. In the magnetic bearing with a redundant structure, the current distribution matrix ***W*** is an important factor that affects the accuracy of EMF. In this paper, we improved the accuracy of the EMF model and took the eight-pole symmetrical radial magnetic bearing as the research object. The corresponding displacement compensation matrices have been calculated for the different coils that fail in the magnetic bearing while the rotor is at the non-equilibrium position. Then, we propose a fault-tolerant control strategy that includes displacement compensation. The rigid body dynamics model of the rotor, supported by magnetic bearings with redundant structures, is established. Moreover, to verify the effectiveness of the proposed control strategy, we combined the rigid body dynamics model of the rotor with a fault-tolerant control strategy, and the corresponding simulation has been carried out. In the case of disturbance force and some coils fail in magnetic bearing and compared with the fault-tolerant control that absents the displacement compensation factors. The simulations demonstrate the disturbance rejection of magnetically levitated rotor system can be enhanced. The robustness of the rotor has been improved with the fault-tolerant control strategy proposed in this paper.

## 1. Introduction

Magnetic bearings are considered to be superior to conventional bearings because of no physical contact, low rotation friction, high speed, and long life [1,2,3]. They are the key components in aero engines, turbine generators, energy storage flywheels, etc. Some advanced control methods were proposed and implicated in other research areas [4,5]. These approaches have been adopted to design the control strategies for magnetic bearings and improved the robustness of the magnetic suspension rotor system [6] However, any part of the magnetic-levitated bearing system that becomes failure will cause a rotor fall, and the system cannot continue working. Therefore, it is necessary to design a fault-tolerant magnetic bearing to improve the reliability of the magnetic-levitated bearing system under extreme conditions [1,7,8].

Considering the fluxes in heteropolar magnetic bearings are strongly coupled, the magnetic bearings with redundant structures and fault-tolerant control are proposed. The fault-tolerant control can support continued operation of the magnetic bearings when its power amplifiers or coils suddenly fail. Maslen and Meeker et al. [9] have proposed a generalized bias current linearization method for heteropolar magnetic-levitated bearings. They designed a current distribution matrix to obtain the current in the remaining coils. Therefore, while some coils failed, the desired bearing force of the *x* and *y* directions can still be provided using the redistribution of the currents in other normal coils. Based on this method, the magnetic bearings can continually support the rotor with some failed coils. The bias current linearization method and current distribution matrix approach have provided a theoretical basis for the fault-tolerant control of redundant magnetic bearings. Na and Palazzolo [10] optimized the fault-tolerant control of magnetic bearings and used the Lagrange multiplier approach to calculate the current distribution matrix and analyzed the position stiffness and voltage stiffness of the fault-tolerant magnetic bearings. They used the simulation approach to verify that the current distribution matrix can achieve the rotor support reconstruction under some coils. Moreover, the corresponding experimental verification was carried out in the flexible rotor, and the result demonstrates that the rotor remained levitated before and after the failure of a coil [11]. Several researchers have designed the fault-tolerant magnetic bearing system for a turbo-molecular vacuum pump. Noh [12] has described the fault-tolerant magnetic bearing system for the eight-pole symmetrical radial magnetic bearing and applied the fault-tolerant control on a turbo-molecular vacuum pump. The result shows that the rotor can remain levitated even with three simultaneously failing poles while at the rotor speed of 4200 rpm. The precise mathematical model of magnetic bearings is significant to designing the corresponding controller, so researchers consider the magnetic leakage and edge and eddy current and reluctance of the ferromagnetic material path factors. Meeker and Maslen [13] defined parameters to describe the magnetic leakage and edge and eddy current effects and established a more accurate magnetic-levitated bearing model. They verified it by experiments. Na and Palazzolo [14] constructed the magnetic bearing model that includes the reluctance of the ferromagnetic material path and the modeling error due to magnetic leakage. They defined a compensation coefficient to make up for errors. Na [15,16] designed magnetic-levitated bearing fault-tolerant controllers based on force invariance or flux invariance theory. However, the work mentioned above is related to the heteropolar magnetic-levitated bearings. According to the feature of homopolar magnetic-levitated bearings, Cheng [17] proposed the flux compensation strategy within one pole pair and the magnetic force compensation strategy between adjacent magnetic poles and discussed the fault-tolerant control approaches with the current safety factor. Meeker [18] proposed a generalized unbiased control strategy for radial magnetic bearings. He extended the method of unbiased control for three-pole radial magnetic bearings to a generalized unbiased control strategy that encompasses bearings with an arbitrary number of poles. Due to neglect of the motion of the rotor in its non-equilibrium position, there exist errors in the EMF model using the current distribution matrix. X. Cheng and Baixin Cheng [19] have calculated the displacement compensation matrices with no failed coiled in the redundant magnetic bearings and added the compensation matrices to the current distribution matrix. The result showed it could improve the accuracy of the electromagnetic force. The generalized bias current linearization needs a complicated calculation process, David Meeker and Eric Maslen [20] proposed a novel method to simplify the process. Still, it only applies to stators with an even number of evenly spaces poles of equal area. The selection of bias current coefficient is important to the fault-tolerant control of magnetic bearings. Inappropriate bias current coefficient value can make the magnetic bearing unable to produce desired force, so Xin Cheng and Shuai Deng [21] proposed an optimal algorithm to obtain the reasonable bias current coefficient value. Numerical verifications demonstrate the effectiveness of the proposed approaches in magnetic bearings with different redundant structures. Meanwhile, Xin Cheng and Shuai Deng [22] designed a fault-tolerant magnetic bearing control system combined with a novel fault-diagnosis of actuators, and the corresponding experimental verification was carried out. Cui [23] combined the bias current linearization theory with the coordinate transformation of position sensors, designed the fault-tolerance of position sensors in the magnetic bearings with redundant structures. Li M H, Palazzolo A B, Kenny A, et al. [24] summarizes the development of homopolar magnetic bearings. They introduced the fault-tolerant theory of magnetic bearing and the generalized bias current linearization theory of magnetic bearings. Wu B [25] used the generalized bias current linearization theory to design a fault-tolerant control scheme. Diagnosis of fault information is an essential task in the fault tolerance of magnetic bearings with redundant structures. For the magnetic bearing coils, some research proposed some coils fault diagnosis algorithms [26,27,28,29,30,31,32]. The most typical method that is applied on magnetic bearings was proposed by Nagel L, Galeazzi R, Voigt AJ, et al. [32].

In this paper, for the faults of some coils in magnetic bearings with redundant structures, the fault-tolerant controller that is based on the theory of improving the accuracy of the EMFs model is proposed. Then we constructed a simulation model with the dynamics of 4-DOF magnetically-levitated rotor and fault-tolerant control that proposed in this paper. Compared with the existing fault-tolerant controller, the proposed controller added the displacement compensation factors to the current distribution controller. The advantages of the proposed controller can improve the output accuracy of electromagnetic force of magnetic bearing, and the linear interval of the electromagnetic force is increased. The numerical results demonstrated that the proposed controller in this paper could reduce the maximum current of coils, effectively suppressing the disturbance force, and improving the robustness of the whole system.

## 2. Generalized Bias Current Linearization of Magnetic Bearings

**Figure 1:**
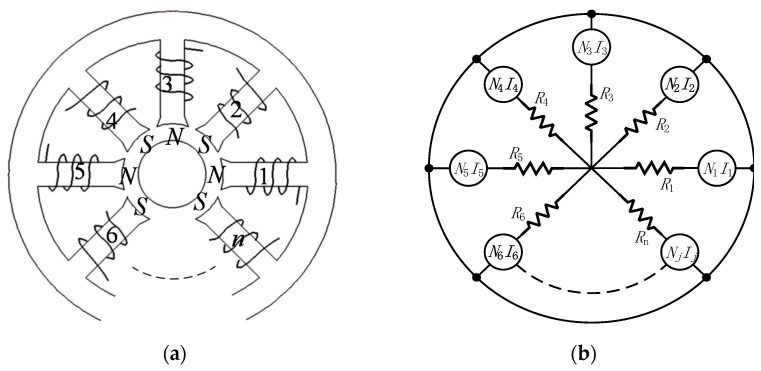
*n* pole bearing arrangement (**a**) and equivalent magnetic circuit (**b**).

For the radial magnetic bearing with *n* pole heteropolar redundant structures depicted in Figure 1a, the equivalent magnetic circuit model can be described by Figure 1b. The only sources of magnetic excitation in the bearing are the coils that are wound on each pole, and virtually all of the circuit reluctance is due to the air gap associated with each pole.

The relationship between the coil current and the magnetic flux can be written as

(1) $$R_{j}\phi_{j}-R_{j+}{}_{1}\phi_{j+1}=N_{j}I_{j}-N_{j+1}I_{j+1},j=1,2,\cdots ,n-1$$

(2) $$R_{j}=\frac{g_{j}}{\mu_{0}A_{j}}$$

where *R_j_* is the reluctance of the *j*th pole gap, *g_j_* is the length of the *j*th air gap, *μ*_0_ is the permeability of vacuum, *n* is the number of poles, and *A_j_*, $\phi_{j}$, *N_j_*, and *I_j_* represent the area, magnetic flux, turns of the coil, and current in the *j*th coil, respectively.

**Figure 2:**
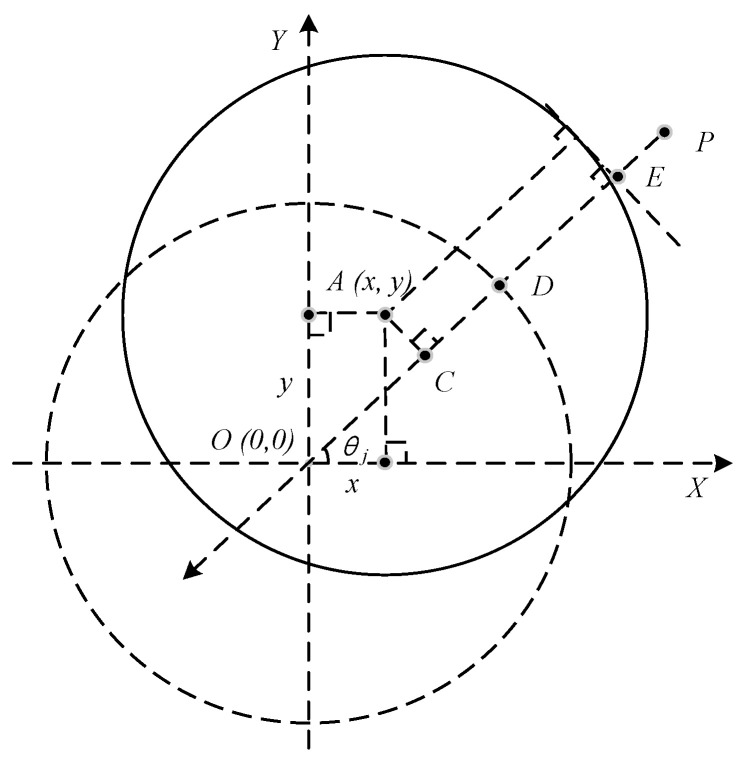
Air gap vs. rotor position.

The air gap *g_j_* is variable while the rotor center is away from its equilibrium position. As shown in Figure 2, the relationship between the *g_j_* (*PE*) and *g*_0_ (PD), *θ_j_* in the coordinate system can be described as Equation (3), when the rotor center at the *A*(*x*, *y*), the air gap *g_j_* can be calculated by Equation (4).

(3) PE=PD−DE=PD−(r−CD)=PD−OC

(4) $$g_{j}=g_{0}-x\cos\theta_{j}-y\sin\theta_{j}$$

The flux conservation is described as

(5) $$\sum_{j=1}^{n}\phi_{j}=0$$

Therefore, the Equations (1)–(5) can be rearranged as

(6) $$\begin{array}{l} \left[\begin{array}{ccccc} R_{1} & -R_{2} & 0 & \cdots & 0\\ 0 & R_{2} & -R_{3} & \ddots & \vdots \\ \vdots & \ddots & \ddots & \ddots & 0\\ 0 & \cdots & 0 & R_{n-1} & -R_{n}\\ 1 & 1 & \cdots & 1 & 1 \end{array}\right]\left[\begin{array}{c} \phi_{1}\\ \phi_{2}\\ \vdots \\ \phi_{n-1}\\ \phi_{n} \end{array}\right]\\ =\left[\begin{array}{ccccc} N_{1} & -N_{2} & 0 & \cdots & 0\\ 0 & N_{2} & -N_{3} & \ddots & \vdots \\ \vdots & \ddots & \ddots & \ddots & 0\\ 0 & \cdots & 0 & N_{n-1} & -N_{n}\\ 0 & 0 & \cdots & 0 & 0 \end{array}\right]\left[\begin{array}{c} I_{1}\\ I_{2}\\ \vdots \\ I_{n-1}\\ I_{n} \end{array}\right] \end{array}$$

and it is rewritten by the matrix form as

(7) RΦ=NI

Assuming that the magnetic flux density is uniform in the gap, the relationship (*φ*_j_ = *A*_j_*B*_j_) can be described by Equation (8).

(8) Φ=AB

where ***A*** is the diagonal matrix of the pole area, ***B*** is the magnetic flux density matrix of the air gap.

(9) $$B=A^{-1}R^{-1}NI=VI$$

In [9], the current distribution matrix ***W*** is defined to describe the relationship between the current in each magnetic pole coil of the magnetic bearing and the logic current. As shown in Equation (10).

(10) $$I=W\left[\begin{array}{c} C_{0}\\ i_{x}\\ i_{y} \end{array}\right]=W(0,0)\left[\begin{array}{c} C_{0}\\ \frac{F_{x}(x,y)}{C_{0}}\\ \frac{F_{y}(x,y)}{C_{0}} \end{array}\right]=WI_{c}$$

where $C_{0}$ is is the bias coefficient, and *i_x_* and *i_y_* is the logical controlled currents in the *x* and *y* directions, respectively. The resultant forces in the *x* and *y* directions, respectively, can be described as

(11) $$F_{x}(x,y)=I_{c}{}^{T}W^{T}M_{x}WI_{c}$$

(12) $$F_{y}(x,y)=I_{c}{}^{T}W^{T}M_{y}WI_{c}$$

where

$$\begin{array}{l} D_{x}=\frac{A_{j}}{2\mu_{0}}diag\left[\cos\theta_{j}\right]\\ D_{y}=\frac{A_{j}}{2\mu_{0}}diag\left[\sin\theta_{j}\right] \end{array}$$

*M_x_* and *M_y_* are real symmetric matrices defined as follows,

(13) $$\{\begin{array}{c} M_{x}(x,y)=-V^{T}D_{x}V\\ M_{y}(x,y)=-V^{T}D_{y}V \end{array}$$

Besides, to ensure the linearization of the electromagnetic force, the current distribution matrix ***W***(*x, y*) should be obtained to satisfy Equation (14).

(14) $$\{\begin{array}{c} W^{T}(x,y)M_{x}W(x,y)-Q_{x}=0\\ W^{T}(x,y)M_{y}W(x,y)-Q_{y}=0 \end{array}$$

where the matrices (*Q_x_* and *Q_y_*) are defined as

(15) $$\begin{array}{l} Q_{x}=\left[\begin{array}{ccc} 0 & 0.5 & 0\\ 0.5 & 0 & 0\\ 0 & 0 & 0 \end{array}\right]\\ Q_{y}=\left[\begin{array}{ccc} 0 & 0 & 0.5\\ 0 & 0 & 0\\ 0.5 & 0 & 0 \end{array}\right] \end{array}$$

Through the calculation of the above theory, the EMFs can be linearized, which are written as

(16) $$\{\begin{array}{c} F_{x}(x,y)=C_{0}i_{x}\\ F_{y}(x,y)=C_{0}i_{y} \end{array}$$

## 3. Fault-Tolerant Control Based Improved EMFs Model

**Figure 3:**
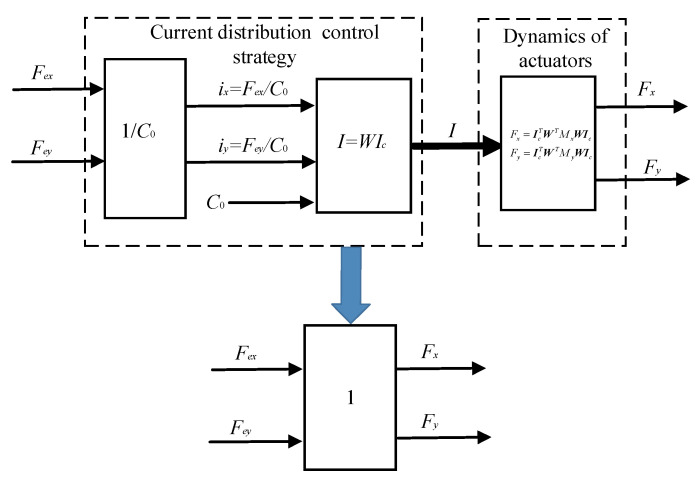
Block diagram of the existing current distribution control strategy.

According to Equations (11) and (12), the EMFs are determined by the structure of magnetic bearings and current distribution matrix ***W***. Based on the dynamics of actuators, the current distribution control of magnetic bearings realize the decoupled and linearized relationship between current and EMFs. Whether or not a failure condition occurs, the system can provide the desired force using the current distribution theory. Therefore, while the current distribution control model is the inverse dynamics model of the actuators, the magnetic bearing system can offer the desired EMFs, as shown in Figure 3, [*F_x_*, *F_y_*] = [*F_ex_*, *F_ey_*]. We can design the distribution control strategy by the current distribution control model, and the distribution control strategy can be used as part of the fault-tolerant control strategy.

**Figure 4:**
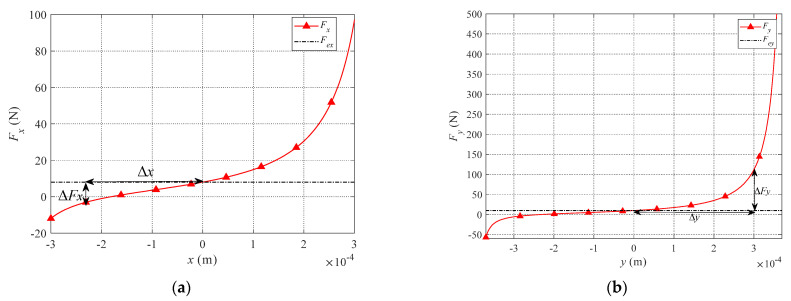
Curves of the relationship between *F_x_* and *x* that is illustrated in (**a**) and relationship between *F_y_* and *y* that is illustrated in (**b**).

Under the condition that the structure of magnetic bearings is determined, there is a quadratic relationship between forces and the air gap, as shown in Figure 4. It will cause serious nonlinear motion of the rotor. Considering the rotor in its equilibrium position, E. H. Maslen and D. C. Meeker [9] have calculated the current distribution matrix ***W*** and the system obtained the desired forces. However, there are errors between actual EMFs *F_x_*, *F_y,_* and expected EMFs *F_ex_*, *F_ey_* while the rotor is in its non-equilibrium position. As indicated in Figure 4, the desired forces are set as *F_ex_* = 8 N, *F_ey_* = 10 N, while the rotor at the equilibrium position (0, 0), the [*F_x_*, *F_y_*] = [*F_ex_*, *F_ey_*]. However, while the center of the motor is away from the equilibrium position [Δx,Δy], the [*F_x_*, *F_y_*] ≠ [*F_ex_*, *F_ey_*], the current distribution control model is no longer equivalent to the inverse dynamics model of the actuators. We define the errors as

(17) $$\Delta F_{x}(\Delta x,\Delta y)=F_{x}(x,y)-F_{ex}(x,y)$$

(18) $$\Delta F_{y}(\Delta x,\Delta y)=F_{y}(x,y)-F_{ey}(x,y)$$

Based on Equations (11) and (12), the errors of forces also can be expressed as

(19) $$\Delta F_{x}(\Delta x,\Delta y)=I_{c}{}^{T}W^{T}\left(\Delta x,\Delta y\right)M_{x}\left(\Delta x,\Delta y\right)W\left(\Delta x,\Delta y\right)I_{c}$$

(20) $$\Delta F_{y}(\Delta x,\Delta y)=I_{c}{}^{T}W^{T}\left(\Delta x,\Delta y\right)M_{y}\left(\Delta x,\Delta y\right)W\left(\Delta x,\Delta y\right)I_{c}$$

The errors between actual EMFs *F_x_*, *F_y_* and expected EMFs *F_ex_*, *F_ey_* will increase the nonlinear characteristics of the rotor and reduce the robustness of the magnetic-levitation rotor.

We can conclude that the EMFs model proposed in [9] has only considered the linearization between the EMFs and the current and neglected the influence between the EMFs and the rotor displacement. However, the EMFs are concerned not only with current but also with rotor displacement, so we can introduce the displacement compensation factors into the current distribution control strategy. It can be a part of the fault-tolerant control strategy.

**Figure 5:**
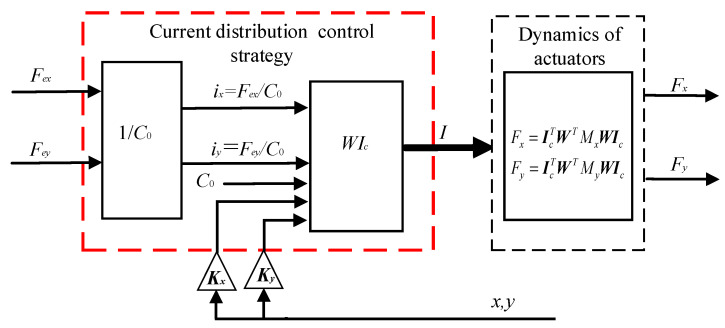
Improved current distribution control strategy proposed in this paper.

The improved current distribution control strategy can be depicted in Figure 5. Compared with the existing fault-tolerant controller, as shown in Figure 3, the difference of the proposed controller in this paper is that the displacement compensation factor ***K_x_*** and ***K_y_*** are added to the current distribution controller.

### 3.1. Improved EMF Model

In Equations (11) and (12), ***M****_x_* and ***M****_y_* represent the characteristics of magnetic bearing with a redundant structure. They are mainly manifested in a magnetic field, reluctance, and other properties. Considering that the air gap between the magnetically-levitated rotor and the protective bearing is generally less than 0.3 mm, the displacement of the rotor center near the equilibrium position cannot be ignored. For the EMFs model in Equations (11) and (12), we can try to expand ***M****_x_* and ***M****_y_* using the Taylor series near the equilibrium position ***M*** (0, 0), as shown in Equations (21) and (22).

(21) $$F_{x}(x,y)=I_{c}{}^{T}W^{T}\left[M_{x}(0,0)+{x\frac{\partial M_{x}}{\partial x}|}_{\begin{array}{c} x=0\\ y=0 \end{array}}+y{\frac{\partial M_{x}}{\partial y}|}_{\begin{array}{c} x=0\\ y=0 \end{array}}\right]WI_{c}$$

(22) $$F_{y}(x,y)=I_{c}{}^{T}W^{T}\left[M_{y}(0,0)+x{\frac{\partial M_{y}}{\partial x}|}_{\begin{array}{c} x=0\\ y=0 \end{array}}+y{\frac{\partial M_{y}}{\partial y}|}_{\begin{array}{c} x=0\\ y=0 \end{array}}\right]WI_{c}$$

The expanded parts of ***M****_x_* and ***M****_y_* can represent the errors, respectively,

(23) $$M_{x}\left(\Delta x,\Delta y\right)=x{\frac{\partial M_{x}}{\partial x}|}_{\begin{array}{c} x=0\\ y=0 \end{array}}+y{\frac{\partial M_{x}}{\partial y}|}_{\begin{array}{c} x=0\\ y=0 \end{array}}$$

(24) $$M_{y}\left(\Delta x,\Delta y\right)=x{\frac{\partial M_{y}}{\partial x}|}_{\begin{array}{c} x=0\\ y=0 \end{array}}+y{\frac{\partial M_{y}}{\partial y}|}_{\begin{array}{c} x=0\\ y=0 \end{array}}$$

Similarly, the first-order Taylor series decomposition of ***W***(*x*, *y*) is performed close to the equilibrium position (0, 0), and an approximation was obtained as follows:

(25) $$W(x,y)\approx W(0,0)+x{\frac{\partial W}{\partial x}|}_{\begin{array}{c} x=0\\ y=0 \end{array}}+y{\frac{\partial W}{\partial y}|}_{\begin{array}{c} x=0\\ y=0 \end{array}}$$

The expanded parts of ***W***(*x*, *y*) have represented the errors, respectively

(26) $$W\left(\Delta x,\Delta y\right)=x{\frac{\partial W}{\partial x}|}_{\begin{array}{c} x=0\\ y=0 \end{array}}+y{\frac{\partial W}{\partial y}|}_{\begin{array}{c} x=0\\ y=0 \end{array}}$$

The partial derivatives of Equation (14) can result in the following equations

(27) $$\{\begin{array}{c} \frac{\partial W^{T}}{\partial x}M_{x}W+W^{T}\frac{\partial M_{x}}{\partial x}W+W^{T}M_{x}\frac{\partial W}{\partial x}=0\\ \frac{\partial W^{T}}{\partial x}M_{y}W+W^{T}\frac{\partial M_{y}}{\partial x}W+W^{T}M_{y}\frac{\partial W}{\partial x}=0 \end{array}$$

(28) $$\{\begin{array}{c} \frac{\partial W^{T}}{\partial y}M_{x}W+W^{T}\frac{\partial M_{x}}{\partial y}W+W^{T}M_{x}\frac{\partial W}{\partial y}=0\\ \frac{\partial W^{T}}{\partial y}M_{y}W+W^{T}\frac{\partial M_{y}}{\partial y}W+W^{T}M_{y}\frac{\partial W}{\partial y}=0 \end{array}$$

The partial derivatives of ***W*** are defined as

(29) $$\{\begin{array}{c} \frac{\partial W}{\partial x}=K_{x}\\ \frac{\partial W}{\partial y}=K_{y} \end{array}$$

Based on the solution theory of the current distribution matrix ***W*** proposed in the literature [6] and the Equations (27) and (28), we can obtain the values of optimal approximation ***W***(*x*, *y*). Through the analysis of the above theory, the unified linearized EMFs model that considers the controlled currents in the coils and rotor displacements can be established as

(30) $$I=\left[W(0,0)+xK_{x}+yK_{y}\right]\left[\begin{array}{c} C_{0}\\ \frac{F_{x}(x,y)}{C_{0}}\\ \frac{F_{y}(x,y)}{C_{0}} \end{array}\right]$$

The ***K_x_*** and ***K_y_*** are defined as the displacement compensation matrices. Compared with Equation (10), the EMFs model described in Equation (30) includes the displacement compensation matrices, and the EMFs model is more accurate than the EMFs model expressed in Equation (10).

### 3.2. Numerical Calculation of the Improved EMF Model

**Figure 6:**
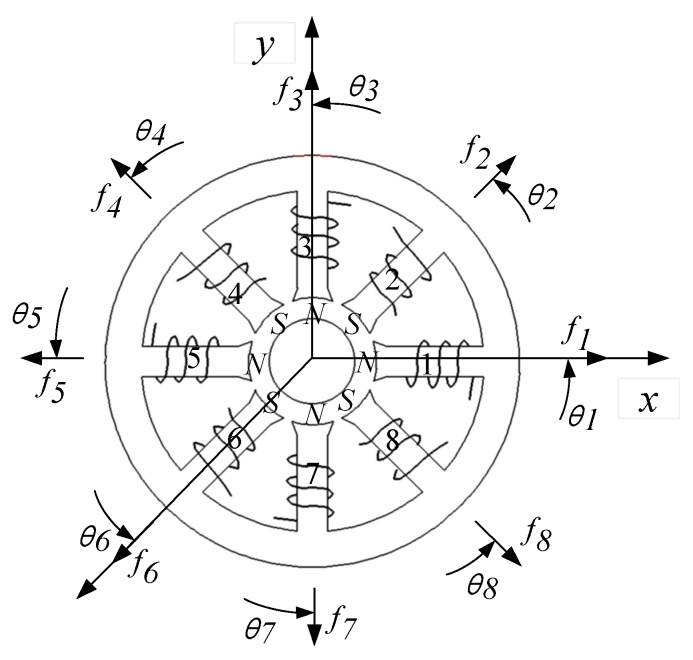
Structural of an eight-pole symmetrical radial magnetic bearing.

Taking an eight-pole symmetrical radial magnetic bearing as an example, its structure is shown in Figure 6, and the parameters are listed in Table 1. While the center of the magnetically-levitated rotor is in a non-equilibrium position, we add the displacement compensation to the current distribution matrix ***W***(*x*, *y*). For the different forms of coils failure in magnetic bearing, the compensation current distributions are calculated respectively.

When there is no failed coil in magnetic bearing, the ***W***(0, 0) is that [9]

(31) $$W\left(0,0\right)=\frac{g_{0}}{4N\sqrt{\mu_{0}A}}\left[\begin{array}{ccc} 2 & 2 & 0\\ -2 & -\sqrt{2} & -\sqrt{2}\\ 2 & 0 & 2\\ -2 & \sqrt{2} & -\sqrt{2}\\ 2 & -2 & 0\\ -2 & \sqrt{2} & \sqrt{2}\\ 2 & 0 & -2\\ -2 & -\sqrt{2} & \sqrt{2} \end{array}\right]$$

Based on the theory of generalized inverse matrix approach, for any fault condition, the corresponding ***W***(0, 0) can be obtained by Equation (32), as shown that

(32) $$W\left(0,0\right)={\left[K^{T}V^{T}VK\right]}^{-1}K^{T}V^{T}VW(0,0)$$

As a consequence, while the 8th pole coil failed, ***W***_8_(0, 0) is expressed as

(33) $$W{\left(0,0\right)}_{8}=\frac{g_{0}}{4N\sqrt{\mu_{0}A}}\left[\begin{array}{ccc} 4 & 2+\sqrt{2} & -\sqrt{2}\\ 0 & 0 & -2\sqrt{2}\\ 4 & \sqrt{2} & 2-\sqrt{2}\\ 0 & 2\sqrt{2} & -2\sqrt{2}\\ 4 & -2+\sqrt{2} & -\sqrt{2}\\ 0 & 2\sqrt{2} & 0\\ 4 & \sqrt{2} & -2-\sqrt{2}\\ 0 & 0 & 0 \end{array}\right]$$

Similarly, while the 6-7-8th pole coils failed, the corresponding ***W***_6-7-8_(0, 0) is expressed as

(34) $$W{\left(0,0\right)}_{6-7-8}=\left[\begin{array}{ccc} -0.7327 & -0.5444 & 0.6410\\ -0.1492 & 0.0821 & 0.9938\\ 0.1289 & -0.0033 & 1.2552\\ -0.2912 & -0.0449 & 1.0012\\ -0.7364 & 0.5522 & 0.5812\\ 0 & 0 & 0\\ 0 & 0 & 0\\ 0 & 0 & 0 \end{array}\right]$$

The air gap between the rotor and the magnetic poles are obtained as

(35) $$g_{j}=g_{0}-x\cos\left[\frac{\left(j-1\right)\pi }{4}\right]-y\sin\left[\frac{\left(j-1\right)\pi }{4}\right]$$

Because the displacement of the magnetically-levitated rotor belongs to the tiny displacement, the current distribution matrix ***W***(*x*, *y*) can be linearized near the equilibrium position. By solving the first-order Taylor series expansion of the current distribution matrix ***W*** while the rotor is at its non-equilibrium position, we can obtain Equation (25). Then, the displacement compensation matrices ***K_x_*** and ***K_y_*** are used to compensate the current distribution matrix ***W***(*x*, *y*) that was obtained. In contrast, the rotor is at its equilibrium position, and at the same time, the relationship between the coils and electromagnetic force is obtained, as shown in Equation (30). We can use equations (Equations (25), (27) and (28)) to obtain the appropriate ***K_x_*** and ***K_y_***. However, there are series of ***K_x_*** and ***K_y_*** satisfying these equations. We can define a cost function as

(36) $$J(W)=\underset{w(x,y)}{\min}{\|W(x,y)-W(0,0)\|}_{2}^{2}$$

In Equation (36), the ***W***(*x*, *y*) minimizes *J* and satisfies the optimal value while the rotor moves to a non-equilibrium position. In this paper, for no failed coil in magnetic bearings, the corresponding solutions of ***K_x_*** and ***K_y_*** are obtained as shown in Equation (37).

(37) $$\begin{array}{c} K_{x}=\left[\begin{array}{ccc} \begin{array}{c} -1083.8774\\ \;766.4147\\ \;0\\ -766.4147\\ \;1083.8774\\ -766.4147\\ \;0\\ \;541.9387 \end{array} & \begin{array}{c} -1083.8774\\ \;541.9387\\ \;0\\ \;541.9387\\ -1083.8774\\ \;541.9387\\ \;0\\ \;541.9387 \end{array} & \begin{array}{c} \;0\\ \;541.9387\\ \;0\\ -541.9387\\ \;0\\ \;541.9387\\ \;0\\ -541.9387 \end{array} \end{array}\right]\\ K_{y}=\left[\begin{array}{ccc} \begin{array}{c} \;0\\ \;766.4171\\ -1083.8774\\ \;766.4171\\ \;0\\ -766.4171\\ \;1083.8774\\ -766.4171 \end{array} & \begin{array}{c} \;0\\ \;563.6163\\ \;0\\ -563.6163\\ \;0\\ \;563.6163\\ \;0\\ -563.6163 \end{array} & \begin{array}{c} \;0\\ \;563.6163\\ -1083.8774\\ \;541.9387\\ \;0\\ \;541.9387\\ -1083.8774\\ \;541.9387 \end{array} \end{array}\right] \end{array}$$

### 3.3. Design the Fault-Tolerant Controller

**Figure 7:**
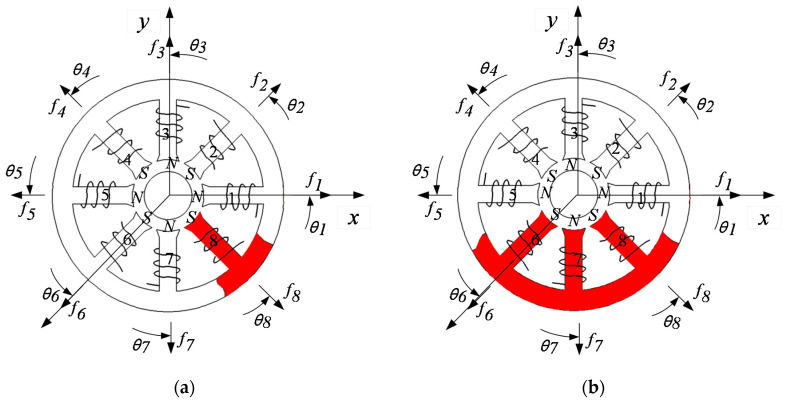
The 8th pole coil and 6-7-8th coils fail in magnetic bearing, marking red in (**a**) and (**b**), respectively.

Considering the 8th pole coil fail or 6-7-8th coils fail in magnetic bearing with redundant structures, marked red color parts as shown in Figure 7, the corresponding displacement compensation matrices ***K***_8***x***_, ***K***_8***y***_ and ***K***_678***x***_, ***K***_678***y***_ are obtained as Equations (38) and (39).

(38) $$\begin{array}{c} K_{8x}=\left[\begin{array}{ccc} \begin{array}{c} -1450.6318\\ \;853.4001\\ \;0\\ -686.1994\\ \;1162.3043\\ -961.6456\\ \;0\\ \;0 \end{array} & \begin{array}{c} -1393.9093\\ \;606.1786\\ \;0\\ \;620.5301\\ -1059.5079\\ \;369.7211\\ \;0\\ \;0 \end{array} & \begin{array}{c} \;526.2552\\ \;509.1354\\ \;0\\ -759.9443\\ -24.6373\\ \;898.6752\\ \;0\\ \;0 \end{array} \end{array}\right]\\ K_{8y}=\left[\begin{array}{ccc} \begin{array}{c} \;0\\ \;961.6456\\ -1116.2304\\ \;686.1994\\ \;0\\ -853.4001\\ \;1450.6318\\ \;0 \end{array} & \begin{array}{c} \;0\\ \;898.6752\\ -24.6373\\ -759.9443\\ \;0\\ \;509.1354\\ \;526.2552\\ \;0 \end{array} & \begin{array}{c} \;0\\ \;369.7221\\ -1059.5079\\ \;620.5301\\ \;0\\ \;606.1786\\ -1393.9093\\ \;0 \end{array} \end{array}\right] \end{array}$$

(39) $$\begin{array}{c} K_{678x}=\left[\begin{array}{ccc} \begin{array}{c} \;1412.1300\\ \;155.6784\\ \;0\\ -286.2799\\ -1370.1311\\ \;0\\ \;0\\ \;0 \end{array} & \begin{array}{c} \;1187.0279\\ -576.7055\\ \;0\\ -406.3168\\ \;1245.4914\\ \;0\\ \;0\\ \;0 \end{array} & \begin{array}{c} -711.2951\\ -1905.2284\\ \;19.4507\\ \;1991.7103\\ \;569.4871\\ \;0\\ \;0\\ \;0 \end{array} \end{array}\right]\\ K_{678y}=\left[\begin{array}{ccc} \begin{array}{c} -592.9683\\ -280.7269\\ -1365.4683\\ \;55.9804\\ -592.9683\\ \;0\\ \;0\\ \;0 \end{array} & \begin{array}{c} \;17.6197\\ -125.9211\\ \;37.4611\\ \;95.7555\\ \;17.6197\\ \;0\\ \;0\\ \;0 \end{array} & \begin{array}{c} \;1165.2429\\ \;156.5345\\ -695.4219\\ \;46.0931\\ \;1165.2429\\ \;0\\ \;0\\ \;0 \end{array} \end{array}\right] \end{array}$$

**Figure 8:**
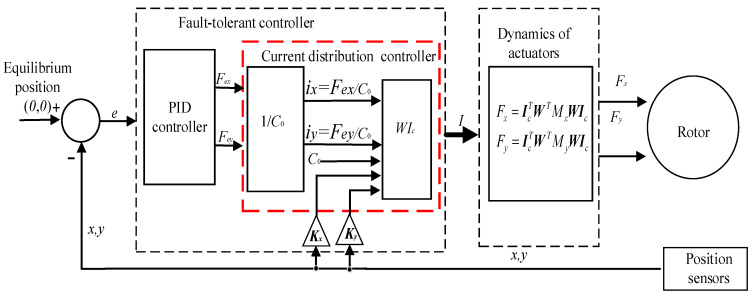
Fault-tolerant control strategy in magnetic bearings with redundant structures.

So far, we can combine Equations (33)–(35) with Equations (37)–(39) to design the fault-tolerant controller suited to magnetic bearings with redundant structures. The entire fault-tolerant controller can be described in Figure 8. The position controller adopts the PID control algorithm.

In Figure 8, the fault-tolerant controller includes the PID controller and current distribution controller. According to the error between the desired rotor position signal and the feedback of the actual rotor position, the PID controller controls the rotor position. For the different form of coils fail in magnetic bearings, the current distribution control strategy realizes the function of redistributing current in the residual coils. Compared with the theory of current configuration proposed in [9], the current distribution algorithm proposed in this paper added the displacement compensation matrices. The essence of the control strategy is the inverse of the controlled object model, and the proposed controller guarantees the linearity of the electromagnetic force. The core of the fault-tolerant controller is to obtain the current distribution matrix ***W***. In this paper, three conditions are considered that include no coils fail, the 8th coil fails, and 6-7-8th coils fails, and the corresponding current distribution matrix ***W*** has been obtained.

## 4. Simulation of Fault-Tolerant Control in the Magnetically-Levitated Rotor

### 4.1. Magnetically-Levitated Rotor Dynamics

In the magnetic bearings with redundant structures, the EMFs generated by each pole of bearing has a nonlinear relationship with the air gap and current. Combining the angle and EMF of each pole, the EMFs of magnetic bearing in the *x* and *y* directions can be written as

(40) $$F_{x}=\left[\begin{array}{cccc} f_{1} & f_{2} & \cdots & f_{8} \end{array}\right]\left[\begin{array}{c} \cos\theta_{1}\\ \cos\theta_{2}\\ \vdots \\ \cos\theta_{8} \end{array}\right]$$

(41) $$F_{y}=\left[\begin{array}{cccc} f_{1} & f_{2} & \cdots & f_{8} \end{array}\right]\left[\begin{array}{c} \sin\theta_{1}\\ \sin\theta_{2}\\ \vdots \\ \sin\theta_{8} \end{array}\right]$$

**Figure 9:**
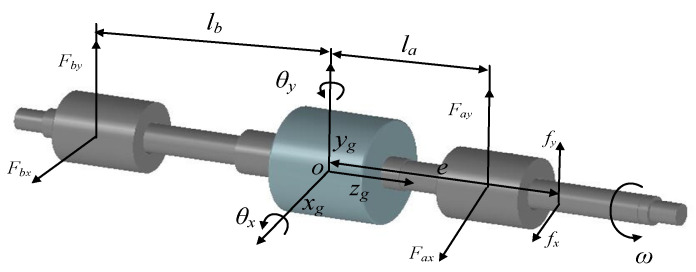
Dynamics of the magnetically-levitated rotor.

In the actual magnetically-levitated rotor system, there are two magnetic bearings with redundant structures. Under rotating conditions, the magnetically-levitated rotor has serious and non-negligible coupling characteristics. Therefore, it is necessary to establish the dynamic model of the rotor. Considering the speed of the rotor is 3000 rpm and lower than the critical bending speed, the rotor, which is levitated by magnetic bearings with redundant structure, can be approximately simplified as a rigid rotor system. Thus, we have established a four-DOF (degree of freedom) magnetically-levitated rotor system shown in Figure 9.

In Figure 9, *F_ax_*, *F_ay,_* and *F_bx_*, *F_by_* denote the EMFs in the *x* and *y* directions of the magnetic bearings A and B. Besides, *l_a_* and *l_b_* express the distances from the bearings A and B to the mass center of the rotor, respectively. *J_x_*, *J_y_* represents the rotor’s inertia moment that is related to the *x*-axis and *y*-axis. The *f_x_* and *f_y_* are the external disturbance forces acting on the rotor in the x and y directions. We use the *e* to describe the distance from the position where the force acts on the rotor to the mass center. Define the rotational speed and mass of the rotor as ω and *m.* Similarly, the *x_g_* and *y_g_* are the displacements of mass center in the *x* and *y* directions, respectively. Neglecting the damping of the rotor and the dynamic model of the rotor can be expressed as

(42) $$\left\{ \begin{array}{l} m{\ddot{x}}_{g}=F_{ax}+F_{bx}+f_{x}\\ m{\ddot{y}}_{g}=F_{ay}+F_{by}+f_{y}\\ J_{x}{\ddot{\theta }}_{x}+J_{z}\omega {\dot{\theta }}_{y}=-l_{a}F_{ay}+l_{b}F_{by}-ef_{y}\\ J_{y}{\ddot{\theta }}_{y}-J_{z}\omega {\dot{\theta }}_{x}=l_{a}F_{ax}-l_{b}F_{bx}+ef_{x} \end{array}\right.$$

where

(43) $$\left\{ \begin{array}{l} x_{g}=\frac{l_{b}}{l}x_{a}+\frac{l_{a}}{l}x_{b}\\ y_{g}=\frac{l_{b}}{l}y_{a}+\frac{l_{a}}{l}y_{b}\\ \theta_{x}=\frac{y_{b}-y_{a}}{l}\\ \theta_{y}=\frac{x_{a}-x_{b}}{l}\\ l=l_{a}+l_{b} \end{array}\right.$$

The *x_a_*, *y_a_*, *x_b_* and *y_b_* are the displacements that correspond to the *x*, *y* directions of radial magnetic bearings A and B. The rotation angles are rotor around the *x*-axis and *y*-axis, respectively. These can be approximated as the displacement of the rotor by Equation (43). By combing the Equations (42) and (43), Equation (42) can be arranged as

(44) $$\left\{ \begin{array}{l} {\ddot{x}}_{a}=-\omega \frac{J_{z}l_{a}}{J_{y}l}({\dot{y}}_{a}-{\dot{y}}_{b})+\;\frac{ml_{a}^{2}+J_{y}}{mJ_{y}}F_{ax}+\frac{J_{y}-ml_{a}l_{b}}{mJ_{y}}F_{bx}+\frac{J_{y}+ml_{a}e}{mJ_{y}}f_{x}\\ {\ddot{x}}_{b}=\omega \frac{J_{z}l_{b}}{J_{y}l}({\dot{y}}_{a}-{\dot{y}}_{b})+\frac{J_{y}-ml_{a}l_{b}}{mJ_{y}}F_{ax}+\frac{ml_{b}^{2}+J_{y}}{mJ_{y}}F_{bx}+\frac{J_{y}-ml_{b}e}{mJ_{y}}f_{x}\\ {\ddot{y}}_{a}=\omega \frac{J_{z}l_{a}}{J_{x}l}({\dot{x}}_{a}-{\dot{x}}_{b})+\;\frac{ml_{a}^{2}+J_{x}}{mJ_{x}}F_{ay}+\frac{J_{x}-ml_{a}l_{b}}{mJ_{x}}F_{by}+\frac{J_{x}+ml_{a}e}{mJ_{x}}f_{y}\\ {\ddot{y}}_{b}=-\omega \frac{J_{z}l_{b}}{J_{x}l}({\dot{x}}_{a}-{\dot{x}}_{b})+\frac{J_{x}-ml_{a}l_{b}}{mJ_{x}}F_{ay}+\;\frac{ml_{b}^{2}+J_{x}}{mJ_{x}}F_{by}+\frac{J_{x}-ml_{b}e}{mJ_{x}}f_{y} \end{array}\right.$$

Based on the state equations as shown in Equation (44), the state variable is selected as

(45) $$X={\left[x_{1},\;x_{2},\;x_{3},\;x_{4},\;x_{5},\;x_{6},\;x_{7},\;x_{8}\right]}^{T}={\left[x_{a},\;x_{b},\;y_{a},\;y_{b},\;\;{\dot{x}}_{a},\;{\dot{x}}_{b},\;{\dot{y}}_{a},\;{\dot{y}}_{b}\right]}^{T}$$

Define the input variable is that

(46) $$U={\left[u_{1},\;u_{2},\;u_{3},\;u_{4}\right]}^{T}={\left[F_{ax},\;F_{bx},\;F_{ay},\;F_{by}\right]}^{T}$$

and output variable is

(47) $$Y={\left[y_{1},\;y_{2},\;y_{3},\;y_{4}\right]}^{T}={\left[x_{a},\;x_{b},\;y_{a},\;y_{b}\right]}^{T}$$

As a consequence, the state equation $\dot{x}=f(x,u)$ can be rewritten as

(48) $$\left\{ \begin{array}{l} \dot{X}=AX+B_{1}U+B_{2}F;\;X(t_{0})=X_{0}\\ Y=CX+DU \end{array}\right.$$

where

$$A=\left[\begin{array}{cccccccc} 0 & 0 & 0 & 0 & 1 & 0 & 0 & 0\\ 0 & 0 & 0 & 0 & 0 & 1 & 0 & 0\\ 0 & 0 & 0 & 0 & 0 & 0 & 1 & 0\\ 0 & 0 & 0 & 0 & 0 & 0 & 0 & 1\\ 0 & 0 & 0 & 0 & 0 & 0 & -\omega \frac{J_{z}l_{a}}{J_{y}l} & \omega \frac{J_{z}l_{a}}{J_{y}l}\\ 0 & 0 & 0 & 0 & 0 & 0 & \omega \frac{J_{z}l_{b}}{J_{y}l} & -\omega \frac{J_{z}l_{b}}{J_{y}l}\\ 0 & 0 & 0 & 0 & \omega \frac{J_{z}l_{a}}{J_{x}l} & -\omega \frac{J_{z}l_{a}}{J_{x}l} & 0 & 0\\ 0 & 0 & 0 & 0 & -\omega \frac{J_{z}l_{b}}{J_{x}l} & \omega \frac{J_{z}l_{b}}{J_{x}l} & 0 & 0 \end{array}\right],B_{1}=\left[\begin{array}{cccc} \frac{ml_{a}^{2}+J_{y}}{mJ_{y}} & \frac{J_{y}-ml_{a}l_{b}}{mJ_{y}} & 0 & 0\\ \frac{J_{y}-ml_{a}l_{b}}{mJ_{y}} & \frac{ml_{b}^{2}+J_{y}}{mJ_{y}} & 0 & 0\\ 0 & 0 & \frac{ml_{a}^{2}+J_{x}}{mJ_{x}} & \frac{J_{x}-ml_{a}l_{b}}{mJ_{x}}\\ 0 & 0 & \frac{J_{x}-ml_{a}l_{b}}{mJ_{x}} & \frac{ml_{b}^{2}+J_{x}}{mJ_{x}} \end{array}\right]$$

$$B_{2}=\left[\begin{array}{cc} \frac{J_{y}+ml_{a}e}{mJ_{y}} & 0\\ \frac{J_{y}-ml_{b}e}{mJ_{y}} & 0\\ 0 & \frac{J_{x}+ml_{a}e}{mJ_{x}}\\ 0 & \frac{J_{x}-ml_{b}e}{mJ_{x}} \end{array}\right],\;F=\left[\begin{array}{c} f_{x}\\ f_{y} \end{array}\right],\;C=\left[\begin{array}{cccccccc} 1 & 0 & 0 & 0 & 0 & 0 & 0 & 0\\ 0 & 1 & 0 & 0 & 0 & 0 & 0 & 0\\ 0 & 0 & 1 & 0 & 0 & 0 & 0 & 0\\ 0 & 0 & 0 & 1 & 0 & 0 & 0 & 0 \end{array}\right]$$

### 4.2. Simulation Verification

**Figure 10:**
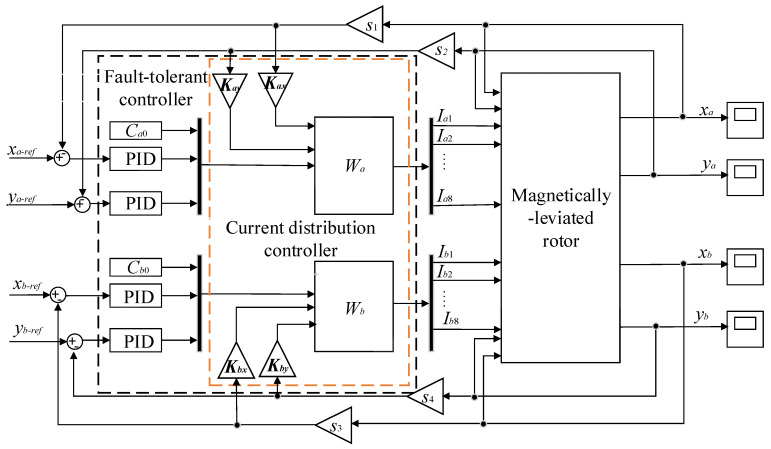
Control block diagram of the entire system.

In this section, we combined the fault- controller, which includes the displacement compensation with the dynamic model of the magnetically-levitated rotor, and carried out the simulation. Then the corresponding simulation results were compared with the results while there is no displacement compensation in the fault-tolerant control strategy proposed in [6]. The control structure is designed as shown in Figure 10 and is simulated based on Matlab/Simulink. In Figure 10, the ***W****_a_* and ***W****_b_* represent the current distribution control of magnetic bearings A and B, respectively. The *x* and *y* directions of bearings A and B are adopted PID controllers to realize position control. For each degree of freedom, the corresponding parameters of the PID controller are the same, and *k_p_* = 10; *k_i_* = 4; *k_d_* = 0.01. The *s*_1_, *s*_2_, *s*_3_ and *s*_4_ represent sensor gain of each degree of freedom of the rotor and *s*_1_ = *s*_2_ = *s*_3_ = *s*_4_ = 5000 V/m. The *C_a_*_0_ and *C_b_*_0_ represent the bias coefficient of magnetic bearings A and B, respectively.

For the rotor, we define *l_a_ =* 0.078 m, *l_b_ =* 0.051 m and *J_x_ =* 1.229 × 10^−3^ Kg·m^2^, *J_y_ =* 1.229 × 10^−3^ Kg·m^2^, *J_z_ =* 5.27 × 10^−5^ Kg·m^2^, *e* = 0.01 m. The reasonable initial state of the rotor is very important for the initial response of the rotor. Otherwise, it will make the rotor unable to reach a stable state. As a consequence, the initial state displacements of the rotor are defined as *x_a_*_0_ = *x_b_*_0_ = −2 × 10^−4^ m, *y_a_*_0_ = *y_b_*_0_ = − 3 × 10^−4^ m, and the *C_a_*_0_ = *C_b_*_0_ = 4. At the initial state, there is no coil fail in the bearings A and B, and the whole time of simulation is 2 s. At the time 0s, the step signal final value of 0 is applied in the *x_a_* direction and other three degrees of freedom. Then, the 8th pole coil of bearing A fails at the time 0.5 s. Finally, the 6-7-8th coils of bearing A fail at the time 1 s. In the simulation, the periodic pulse and the sine external interference are taken to verify the effectiveness of the fault-tolerant control strategy proposed in this paper.

(1) Periodic pulse disturbance test

The external interference forces *f_x_* = *f_y_* = 60 N and sustain time of 0.5 ms are simulated by the 0.2 s periodic pulse in this paper.

**Figure 11:**
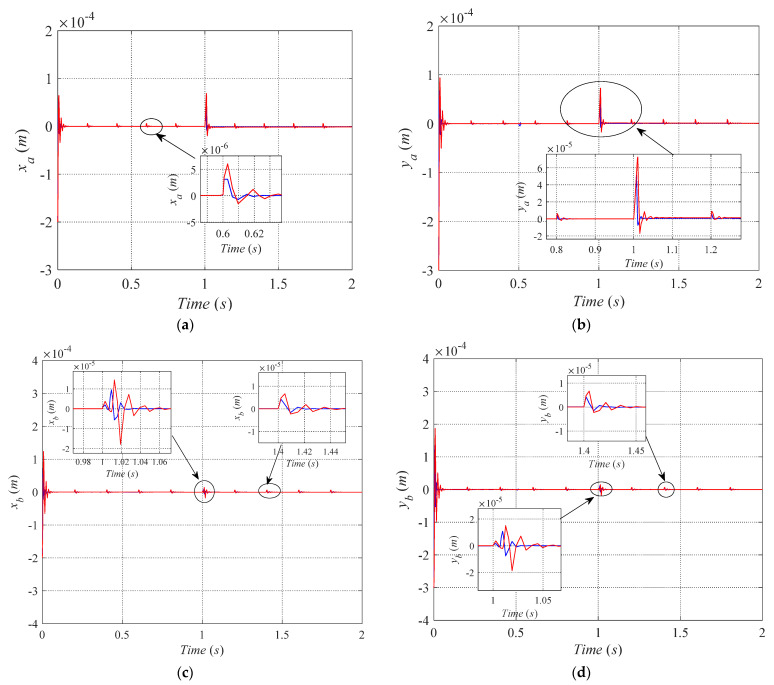
The rotor’s disturbance recovery performance under existing fault-tolerant control by red curve and in this paper by the blue curve. The trajectories of *x_a_, y_a,_ x_b_* and *y_b_* are respectively illustrated in (**a**–**d**) that the periodic pulse disturbance is considered.

The position controllers adopt the PID control algorithm that is shown in Figure 10. The trajectories of the rotor are convergence while the rotor is at the equilibrium position. We can conclude that the proposed approach guarantees the stability of the system. As illustrated in Figure 11, the red and blue curves denote the trajectories of the magnetically-levitated rotor under the fault-tolerant control strategy proposed in [9] and this paper, respectively. As shown in Figure 11a–d, we can see that the maximum fluctuation of trajectories of the magnetically-levitated rotor arises at the time of the 6-7-8th coils fail. Under the control strategy proposed in [9], Figure 11b demonstrates the maximum offset of displacement of the magnetically-levitated rotor is about 6.5 × 10^−5^ m, and the rotor returns to the equilibrium position after about 0.06 s. However, while the magnetically-levitated rotor system under control strategy proposed in this paper, the maximum offset of rotor displacement is about 4.5 × 10^−5^ m, and the rotor returns to the equilibrium position after about 0.02 s. It can be seen that compared with the control strategy proposed in [9], the maximum offset of the rotor displacement is reduced by around 30% using the control strategy proposed in this paper and the time to return to the equilibrium position is around 33% of the control strategy proposed in this paper. The same characteristics exist in *x_b_* and *y_b_* directions, as shown in Figure 11c, d. We can conclude that the fault-tolerant control strategy proposed in this paper has a good inhibiting effect on the periodic pulse disturbance and makes the system’s anti-jamming be improved.

(2) Sinusoidal disturbance test

**Figure 12:**
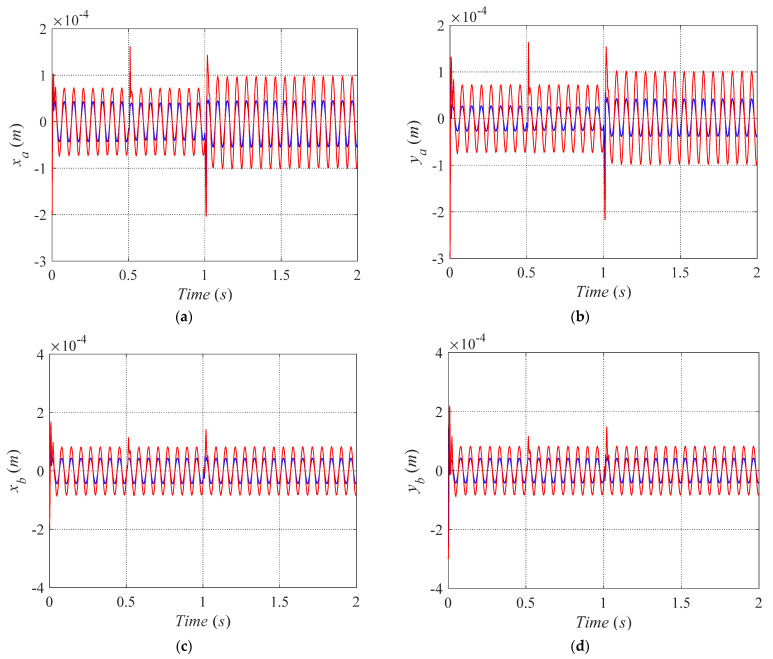
Trajectories of the magnetically-levitated rotor in four degrees of freedom directions under the existing fault-tolerant control by red curve and proposed in this paper by blue curve. The curves of *x_a_, y_a,_ x_b_* and *y_b_* are respectively illustrated in (**a**–**d**) that the sinusoidal disturbance is considered.

The disturbance forces are defined as *f_x_* = *f_y_* = 10 sin(100 *t*) N. During the whole simulation time, there is no coil fail in bearing B. The trajectories of the magnetically-levitated rotor in four degrees of freedom directions are shown in Figure 12. Similarly, the red curve indicates the displacement response of the rotor under the fault-tolerant control strategy proposed in [9]. Meanwhile, the blue curve denotes the displacement response of the rotor under the fault-control strategy proposed in this paper.

As shown in Figure 12, the position where the disturbance forces are applied is close to the magnetic bearing A, and the amplitudes of *x_a_* and *y_a_* are larger than *x_b_* and *y_b_*. Figure 12 demonstrates that under the case of fault-tolerant control mode and external sinusoidal disturbance force, the suspension state of the rotor can be reconstructed while some coils failed in the magnetic bearing A. At the time of 0.5 s, the 8th pole coil of bearing A fails, and the rotor maintains the suspension state. Meanwhile, the fluctuation of the displacement amplitudes in the *x_a_* and *y_a_* directions of the rotor are maintained at about ±0.7 × 10^−4^ m. When the 6-7-8th coils of bearing A fail at the time 1 s, the rotor also realized the reconstruction of the suspended state. However, the amplitudes of *x_a_* and *y_b_* have increased.

On the one hand, while the magnetically-levitated rotor system under control strategy proposed in [9], the value of *x_a_* fluctuates in ±1.0 × 10^−4^ m, and *y_a_* fluctuates in ±1.0 × 10^−4^ m which are shown as red curve in Figure 12a,b. Besides, the value of *x_a_* and *x_b_* fluctuates in about ±1.5 × 10^−4^ m while the 6-7-8th coils of bearing A fail at the time 1 s. On the other hand, while the magnetically-levitated rotor system under control strategy proposed in this paper, the value of *x_a_* fluctuates in ±0.4 × 10^−4^ m, and *y_a_* fluctuates in ±0.4 × 10^−4^ m which are shown as the blue curve in Figure 12a,b. Similarly, the value of *x_a_* and *x_b_* fluctuates in about ±0.4 × 10^−4^ m while the 6-7-8th coils of bearing A fail at time 1 s. Compared with the maximum rotor displacement fluctuation of *x_a_*, *y_a_*, the system under the fault-tolerant control strategy proposed in [9], the amplitudes of *x_a_* and *y_a_* reduce about 60% by using the proposed control strategy in this paper.

Because the position where the disturbance forces are applied is far from the magnetic bearing B, and there is on coils fail in the magnetic bearing B in the whole simulation process, the fluctuating amplitudes of *x_b_* and *y_b_* are smaller *x_a_* and *y_a_* as shown in Figure 12c,d. In contrast, the magnetically-levitated rotor system under control strategy that proposed in [9], the values of *x_b_*, *y_b_* fluctuates in about ± 0.8 × 10^−4^ m. However, the values of *x_b_*, *y_b_* become fluctuates in about ±0.4 × 10^−4^ m while the system adopts the control strategy proposed in this paper. Compared with the control strategy proposed in [9], the maximum offset of the *x_b_*, *y_b_*, is reduced by around 50% using the control strategy proposed in this paper. The magnetic bearing system adopts the proposed control strategy maintaining good anti-disturbing capability when exposed to a sudden periodic pulse disturbance and sinusoidal disturbance. The results demonstrate that compared with the existing fault-tolerant controller, the proposed controller has improved the robustness of the rotor.

**Figure 13:**
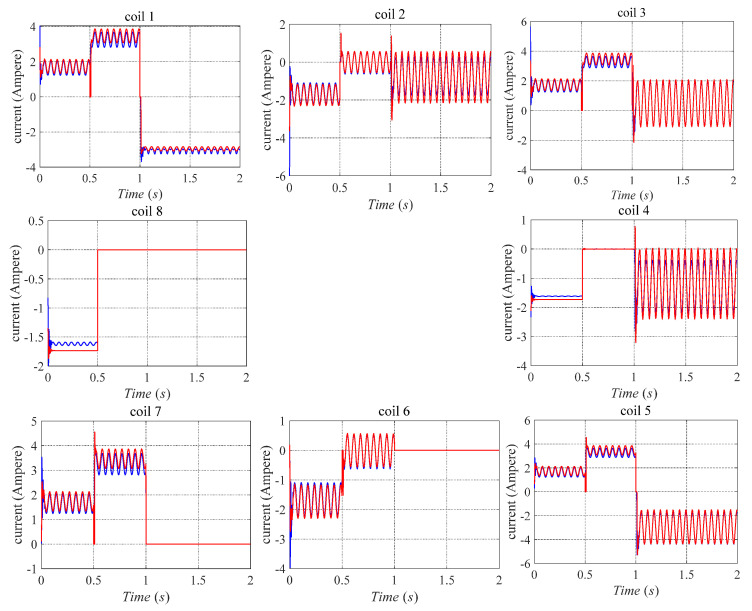
Current inputs of bearing A for a while the system under existing fault-tolerant control by red curve and proposed in this paper by the blue curve.

The transient response of the current inputs to bearing A for the whole simulation time is shown in Figure 13. Similarly, the red curve indicates the coils’ current response in bearing A under the fault-tolerant control strategy proposed in [9]. Meanwhile, the blue curve denotes the coils’ current response in bearing A under the fault-tolerant control strategy proposed in this paper. Spikes occur when the 8th and 6-7-8th coils suddenly failed. However, compared with the control strategy proposed in [9], the current value decreases using the control strategy proposed in this paper. It can be seen that the control strategy proposed in this paper can effectively reduce the value of the control current and reduce the power consumption of the system.

## 5. Conclusions

For the heteropolar magnetic bearing with a redundant structure, when the rotor is in its equilibrium position, the relationship between the EMF and current can be linearized by the theory of generalized bias current linearization. However, the coils’ failure and reconstruction of rotor suspension will increase the error between rotor center position and equilibrium position. Thus, by solving the Taylor series expansion equation of ***W***(*x*, *y*) in the non-equilibrium position, introducing a set of displacement compensation matrices corresponding to the coils failed. Based on the dynamic model of the magnetically-levitated rotor, the effectiveness of the proposed controller was simulated. The numerical results demonstrated that the fault-tolerant control strategy proposed in this paper could have better robustness to the external disturbance than the existing fault-tolerant control scheme. Consequently, we believe that the proposed fault-tolerant control strategy can improve the robustness of the magnetically-levitated rotor, especially while some coils fail in the magnetic bearings and reduce the system’s power consumption. Furthermore, the vibration signals can more directly reflect the vibration suppression effect of the rotor. Considering that the current research work does not consider vibration signals, further research work will use the vibration signals to evaluate the controller’s performance.

## Figures and Tables

**Table 1:** Units for Magnetic Properties.

Structure Parameter	Value	Unit
Pole area, *A*_0_	5.4 × 10^−5^	m^2^
Turns per coil, *N*	56	/
Pole initial gap *g*_0_	4 × 10^−4^	m
Pole angle, *θ_j_*	(*j* − 1) *π*/4	rad
Saturation magnetic-flux density, *B_sat_*	1.2	T
Rotor weight, *m*	0.8	kg

## Data Availability

Not applicable.
